# Isolation, identification, and antibiogram studies of *Escherichia coli* from ready-to-eat foods in Mymensingh, Bangladesh

**DOI:** 10.14202/vetworld.2022.1497-1505

**Published:** 2022-06-15

**Authors:** Fateha Akther Ema, Rifat Noor Shanta, Md. Zaminur Rahman, Md. Ariful Islam, Mst. Minara Khatun

**Affiliations:** Department of Microbiology and Hygiene, Bangladesh Agricultural University, Mymensingh, Bangladesh

**Keywords:** antibiogram profile, *Escherichia coli*, prevalence, ready-to-eat foods

## Abstract

**Background and Aim:**

Ready-to-eat (RTE) foods are widely used at home, restaurants, and during festivals in Bangladesh. So it is very important to investigate possible microbial contamination in RTE foods. Therefore, this study aimed to determine the total coliform count (TCC), isolate, identify, and characterize the *Escherichia coli* in RTE foods. The antimicrobial sensitivity of *E. coli* obtained from RTE foods was also performed using 12 commonly used antibiotics.

**Materials and Methods:**

A total of 100 RTE food samples were collected aseptically and comprised of ten samples each: Burger, pizza, sandwich, chicken roll, chicken meat loaf, chicken fry, salad vegetable, ice-cream, yogurt, and milkshake sold in Mymensingh City. Samples were inoculated onto Eosin methylene blue agar and incubated at 37°C for 24 h. Isolation and identification of bacteria were performed based on cultural, staining, and biochemical properties, followed by a polymerase chain reaction.

**Results:**

The TCC in Chicken meat loaf, burger, pizza, sandwich, salad vegetable ice-cream, and yogurt samples were 3.57 ± 0.96, 3.69 ± 0.08, 3.50 ± 0.60, 2.60 ± 0.20, 4.09 ± 0.29, 4.44 ± 0.25, and 3.14 ± 0.30 mean log colony-forming units ± standard deviation/mL, respectively. The study found a higher prevalence of *E. coli* in RTE salad vegetable products than in RTE meat and milk products. Forty percent of the mixed vegetable salad samples showed positive results for *E. coli*. Whereas *E. coli* prevalence in RTE meat and milk products was 20% and 16.7%, respectively. All the 21 isolates were subjected to antibiotic susceptibility test against 12 different antibiotics. It was observed that 46.1% were susceptible, 16.6% were intermediate, 46.1% were resistant, and 47.6% were multidrug-resistant (MDR) among seven different antibiotic classes. *E. coli* isolates were resistant to cephalexin, ceftazidime, oxytetracycline, and ampicillin and sensitive to gentamycin, followed by kanamycin, ceftriaxone, colistin, and enrofloxacin.

**Conclusion:**

The study revealed that RTE foods are a serious issue from a public health point of view. To achieve a safer level of *E. coli* in RTE foods sold for human consumption, public food outlets must improve hygienic and good production procedures. Moreover, MDR *E. coli* in these foods pose serious public health threats.

## Introduction

Animal-derived foods, such as chicken meat, beef, and milk are high in proteins, which are essential for body growth and development. Foods of animal origin, on the other hand, can operate as a vehicle and medium for the transmission of numerous microorganisms that can cause health problems, disease, and death. Food-borne illnesses are becoming a global public health concern. These microorganisms are responsible for an estimated 48 million illnesses and 3000 fatalities in the United States each year [1]. In contrast, ready-to-eat foods (RTE) do not require any additional preparation, with the exception of warming, and these RTE foods are often eaten raw or cold without any additional heat treatment [2, 3]. Because of rapid population expansion and the modern lifestyle, longer working hours, increased women’s participation in the labor market, and changes in cooking and eating habits, RTE food consumption has surged in recent years. For busy city inhabitants, RTE foods are convenient. Food-borne outbreaks are known to occur as a result of handling, preparing, and marketing these items [3].

RTE meat products are in high demand due to their biological value, reasonable price, agreeable taste, and easy preparation. Meat products are considered excellent sources of high-quality protein, minerals, and vitamins [4]. Moreover, these foods are shelf-stable, flavorful, affordable, and immediately accessible to customers because they do not require a lengthy pre-treatment process [5, 6]. The possibility of *Escherichia coli* to induce health risks occurs mainly during the preparation and storage of contaminated RTE meat [7]. However, because RTE meats have been identified as transporters for food-borne bacteria such as *E. coli*, these foods provide a significant microbiological risk [8]. Thus, food-borne illnesses comprise various diseases responsible for causing morbidity and mortality worldwide [9]. As a result, food-traceability systems, particularly for meat and meat-derived products, are urgently needed to improve the quality of food-processing events and ensure safe food for final consumers [10].

*E. coli* is a widespread species found in the intestines of farm animals, poultry, and humans. The majority of *E. coli* strains are non-pathogenic, but a few are very pathogenic, causing watery and bloody diarrhea; *E. coli* 0157:H7 has been linked to life-threatening diseases such as hemorrhagic colitis (HC), hemolytic uremic syndrome (HUS), and thrombotic thrombocytopenic purpura [1]. *E. coli* have been found to contaminate RTE foods [11]. This species can survive on hands and other surfaces and is readily transferred to foods [12]. As people consume more poultry, milk, and beef, the risk of contracting diseases of animal origin, such as pathogenic *E. coli*, has increased. The major inhabitant of human and animal guts is *E. coli*, a member of the Enterobacteriaceae family. *E. coli* has been identified as indicator species of fecal and enteric pathogen contamination. Although the majority of *E. coli* strains are non-pathogenic, some are known to cause significant human gastrointestinal disorders such as HC and HUS. Shiga toxins (*stx1* and *stx2*), enterohemolysin (*hlyA*), and intimin (*eaeA*) are virulence factors that play a key role in the development of these disorders [13]. There are additional studies focusing on *E. coli* and other pathogens in RTE foods [14].

Antibiotics have been used in human and veterinary medicine for many years to minimize morbidity and mortality and the economic effect of bacterial infections. However, *E. coli* has developed resistance to one or more antibiotics, which has raised public health concerns. The indiscriminate and rising use of antibiotics is linked to the high occurrence of resistant bacteria. Antimicrobials are used in the food production process to prevent and control illnesses, improve growth, and increase feed efficiency in food-producing animals [15]. The use of these antibiotics at low doses for long periods of time to feed animals, for example, can result in the selection and spread of antibiotic resistance to other microbes in the food chain [16].

Nonetheless, plant-based foods, particularly salads, and RTE street foods/meals play a key role in antibiotic resistance transmission and are becoming a serious concern. Various workers have isolated multidrug-resistant (MDR) and extended-spectrum beta-lactamase (ESBL) producing *E. coli* from raw meat, vegetable salad, egg surface, unpasteurized milk, raw fish, and water, indicating major public health concerns [17, 18]. To ensure complete food safety, studies on pathogenic *E. coli* serotypes in RTE foods must be continued. Many foods, particularly those of animal origin and those foods exposed to sewage pollution have been recognized as vehicles for spreading pathogens to humans [19]. Worldwide, treating *E. coli* infections has become complicated due to the increasing prevalence of drug-resistant strains. The emerging resistance found in *E. coli* strains to most antibiotics is a serious threat to consumers’ health [20].

Therefore, this study aimed to determine the total coliform count (TCC), isolate, identify, and characterize the *Escherichia coli* in RTE foods in Mymensingh, Bangladesh. The antimicrobial sensitivity of *E. coli* obtained from RTE foods was also performed using 12 commonly used antibiotics.

## Materials and Methods

### Ethical approval

This study was approved by Animal Welfare and Experimentation Ethics Committee, Bangladesh Agricultural University, Mymensingh [AWEEC/BAU/2019 (58)].

### Study period and location

The present study was conducted from January 2019 to December 2019 in the Department of Microbiology and Hygiene, Bangladesh Agricultural University (BAU), Mymensingh.

### Sampling

A total of 100 RTE food samples comprising ten samples each of burger, pizza, sandwich, chicken roll, chicken meatloaf, chicken fry, salad vegetable ice-cream, yogurt, and milkshake samples were collected aseptically on a random basis from a different restaurants and fast food shops of BAU campus and Mymensingh city. The samples were immediately transferred to appropriate containers and labeled with an identification mark. The samples were carefully handled, kept in an icebox (4°C) and immediately transported to our laboratory facilities for analysis.

### Processing of sample

10 g of samples were blended in a sterilized mortar and pestle. Then samples were homogenized thoroughly with 90 mL of sterile phosphate-buffered saline (PBS; pH 7.4, Merck KGaA, Germany) solution to make a 10% sample suspension. Tenfold serial dilutions of the sample (10^−1^ to 10^−10^) were prepared as per the recommendation of Trojan *et al*. [20] and the International Organization for Standardization (ISO) [21]. This involved mixing 1 mL of a homogenized sample with 9 mL of sterile PBS. In case of ice-cream, yogurt, and milkshake samples, these were mixed with 9 mL of 0.1% peptone water (Oxoid Ltd, England) and incubated at 37°C for 24–48 h.

### Enumeration of total viable count

Eosin methylene blue (EMB) agar media (HiMedia, India) were prepared according to the manufacturer’s instructions and sterilized by autoclaving for 15 min at 121°C. 0.1 mL of each tenfold dilution was transferred and spread in duplicate onto EMB agar plates (HiMedia) using a micropipette for each dilution. The diluted samples were spread as quickly as possible on the surface of the plate with a sterile spreader. The plates were kept in an incubator at 37°C for 24 h. After incubation, plates exhibiting 30–300 colonies were counted. The average number of colonies in a particular dilution was multiplied by the dilution to obtain the TCC. The TCC was calculated according to ISO method [21]. The results of the TCC were expressed as the number of colony-forming units (CFU) per gram of food sampled. CFU/g was determined using the following formula: Number of colonies × dilution factor/volume of culture plated. In case of RTE food, the CFU were determined.

### Isolation and identification of *E. coli*

Each of the homogenized samples was then transferred into nutrient broth (NB, 5 mL/test tube, HiMedia) and incubated at 37°C for 24 h for enrichment; a small amount of inoculum from NB was streaked in duplicate onto EMB agar plates and incubated at 37°C overnight [22]. The colonies which produced metallic green sheen with dark centered colonies were selected. The isolated colonies were sub-cultured several times to assess the uniformity of colony appearance. Bacteria were identified based on colony characteristics, morphological characteristics by Gram’s staining, sugar fermentation test, and a series of biochemical tests, including methyl red, Voges-Proskauer (V-P), catalase, and coagulase [23].

### Identification of *E. coli* isolates using analytical profile index (API) 20E

A standardized identification system, API 20E (Biomerieux, France), was used for Enterobacteriaceae and other Gram-negative rods **[**23, 24] for the identification of *E. coli*. Pure *E. coli* isolates were sub-cultured on EMB agar and incubated at 37°C for 18–24 h. The identification test of *E. coli* isolates was conducted according to the manufacturer’s protocol. A homogeneous bacterial suspension was obtained for selected colonies using API 20E medium. Both tubes and cupules of API 20E were filled with the inoculated API 20E media. Anaerobiosis was ensured in the arginine dihydrolase, lysine decarboxylase, ornithine decarboxylase, urease and H_2_S production tests by filling the cupules with sterile mineral oil to form a convex meniscus. The incubation boxes were closed and incubated at 37°C for 18 to 24 h. Identification of isolates was performed according to the numerical profile of API 20E observed.

### Polymerase chain reaction (PCR)

The extraction of deoxyribonucleic acid (DNA) was carried out using the boiling method [24]. To confirm the *E. coli*, PCR assays were used on all 21 isolates. A highly conserved area, 16S rRNA, was chosen for *E. coli* identification; Table-1 represents the primer list used for the PCR assay [25, 26]. The primers ECO-1 Forward GACCTCGTTTAGTTCACAGA and ECO1 Reverse-CACACGCTGACGCTGACCA with an amplicon size of 585 bp [27] were used with the nucleotide sequence (5’-3’). For a single sample, the PCR reaction mixture was 20:l, with 5:l of Ribonuclease-free water, 10:l of PCR master mixture (Thermo Scientific, EU), 3:l of genomic DNA, and 2:l of primer (ARE THE NUMBERS RATIOS OR UL OF VOLUME?). The PCR amplification was done by initial denaturation at 94°C for 3 min, followed by 35 cycles of denaturation at 94°C for 45 s, annealing at 55°C for 45 s, and extension at 72°C for 60 s. The final extension was at 72°C for 7 min. PCR amplified products were subjected to gel (1% agarose, Takara, Japan) electrophoresis and then exposed to ultraviolet light and any fluorescence (100v for 30 min) visualized in gel documentation system via UV transilluminator (302 nm). To identify Shiga toxin-producing *E. coli*, PCR was also performed using *stx-1* and *stx-2* gene-specific primers.

**Table 1:** Oligonucleotide primers and the PCR program used for amplification of virulence factors in the *Escherichia coli* isolates of ready-to-eat foods.

Primer names	Target genes	Primer sequences (5’-3’)	Size (bp)	References
*EC 16srRNA* (F)	*16srRNA*	5’GACCTCGGTTTAGTTCACAG3’	585	[25]
*EC 16srRNA* (R)		5’CACACGCTGACGCTGACCA3’		
*Stx-1* (F)	*stx-1*	5’CACAATCAGGCGTCGCCAGC GCACTTGCT3’	606	[26]
*Stx-1* (R)		5’TGTTGCAGGGATCAGTCGTA CGGGGATGC3’		
*stx-2* (F)	*stx-2*	5’CCACATCGGTGTCTGTTATTA ACCACACC3’	372	[26]
*stx-2* (R)		5’GCAGAACTGCTCTGGATGCA TCTCTGGTC3’		

### Antibiotic susceptibility test

The Kirby–Bauer disk diffusion method described by Bauer *et al*. [28] was used to perform an antimicrobial susceptibility test on Mueller–Hinton agar (MHA) (HiMedia) using the following antibiotic disks: Cephalexin (30 μg/disk), ampicillin (10 μg/disk), streptomycin (10 μg/disk), gentamycin (10 μg/disk), colistin (10 μg/disk), enrofloxacin (5 μg/disk), nalidixic acid (30 μg/disk), ceftriaxone (30 μg/disk), ceftazi­dime (30 μg/disk), oxytetracycline (30 μg/disk), cefixime (5 μg/disk), and kanamycin (30 μg/disk) (HiMedia). The zone sizes of bacteria were compared with standards for resistance and sensitivity stated by the Clinical and Laboratory Standards Institute [29].

### Phenotypic detection of ESBL producing *E. coli* by double-disk synergy test (DDST)

The DDST was performed to determine the synergy between a disk of amoxicillin-clavulanic acid (AMC) (20/10 µg) and 30 µg disks of each ceftazidime and cefotaxime, aztreonam, and ceftriaxone placed at a distance of 20 mm (center to center) from the AMC disk [30]. The test inoculum (0.5 McFarland turbidity) was spread onto MHA (HiMedia) using a ster­ile cotton swab. A disk of AMC (20 µg amoxicillin + 10 µg clavulanic acid) was placed on the surface of MHA (HiMedia); then 30 μg disks of each cefotaxime, ceftazidime, aztreonam, and ceftriaxone were placed in such a way that each disk was at a distance ranging between 16 and 20 mm from the AMC disk (center to center). The plate was incubated at 37°C overnight. Any distortion or increase in the zone toward the disk of AMC was considered as positive for the ESBL production by Clavulanic acid (provided by the AMC disk) and the subsequent action of the extended-spectrum cephalosporin or aztreonam [31]. If no distortion occurs, then the result is negative for ESBL by the AMC.

### Statistical analysis

The results of TCC of bacteria found in the RTE foods sold at local markets were analyzed for statistical significance using Duncan’s multiple range test (Statistical Package for the Social Sciences, version 11.5, IBM Corp., NY, USA). p < 0.05 was considered to be statistically significant.

## Results

### Prevalence of *E. coli*

Based on cultural, staining, and biochemical characteristics, the overall prevalence of *E. coli* in food was 21% and in RTE meat products 20%, RTE milk products 17%, and salad vegetables 40% (Table-2). The prevalence of *E. coli* in different RTE food samples is presented in Table-3. None of the isolates were found to harbor *stx-1* and *stx-2* genes.

**Table 2:** Overall prevalence of *Escherichia coli* in different types of ready-to-eat food samples.

Food Samples	Meat Samples	Milk Samples	Vegetable Samples	Total
Number of samples tested	60	30	10	100
Number of culture positive samples	12	5	4	21
Prevalence (%)	20	16.66	40	21

**Table 3:** Prevalence of *E. coli* in ready-to-eat food samples.

Name of ready to eat foods	Number of ready to eat foods	Number of Culture positive samples (n)	Number of 16Sr RNA positive samples	*stx-1* positive	*stx-2* positive	Prevalence of *E. coli* (%)	Overall Prevalence of *E. coli* (%)
Chicken roll	10	-	-	-	-	-	21
Chicken meat loaf	10	2	2	-	-	20	
Chicken fry	10	-	-	-	-	-	
Burger	10	3	3	-	-	30	
Pizza	10	5	5	-	-	50	
Sandwich	10	2	2	-	-	20	
Salad vegetables	10	4	4	-	-	40	
Ice-cream		3	3	-	-	30	
Yogurt	10	2	2	-	-	20	
Milk shake	10	-	-	-	-	-	
Total	100	21	21	-	-	21	

*E. coli*=*Escherichia coli*

### Cultural, staining, and biochemical characteristics

*E. coli* creates colonies with a dark metallic sheen on EMB agar, and Gram staining revealed that *E. coli* forms Gram-negative short rods grouped either as solitary, paired or in short chains. Most isolates fermented the six basic sugars, dextrose, maltose, lactose, sucrose, and mannitol, creating acid and gas, while a few fermented all five basic sugars except sucrose.

### Identification of *E coli* using API 20E systems

After incubating the tray at 37°C for 18–24 h, some of the compartments showed a change in color after 24 h, but for some, we had to add reagents before reading. After the addition of reagents, the TDA, and indole test became pink, indicating a positive result and V-P remained colorless, which means a negative result. All individual results were recorded and later matched with API log book or API web for identification.

### Molecular detection

**Figure-1:**
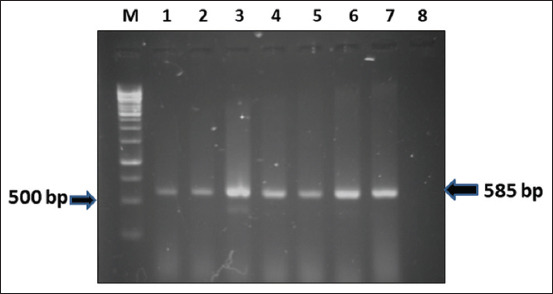
Results of PCR for amplification of *Escherichia coli* 16srRNA gene (585 bp). Lane M: 100 bp DNA marker; Lane 1–7: DNA of 16srRNA positive *E. coli* isolates; Lane 8: Negative control. PCR=Polymerase chain reaction.

The result of PCR assay is presented in Figure-1. The amplified size of PCR product was 585 bp indicated or reconfirmed as *E. coli* isolate in the RTE food samples. None of the isolates were found to amplify *stx-1* and *stx-2* (data not shown).

### Total coliform/*E. coli* count

All ten food items were examined for determination of TCC. Table-4 represents the results of bacteria isolates with their mean values expressed in log10 CFU/mL. From these results, the average *E. coli* count was estimated from different kinds of RTE food and ranged between 2.60 ± 0.20 and 4.50 ± 0.60 CFU ± standard deviation (SD)/mL. The highest number of *E. coli* counts isolated from pizza samples was 4.50 ± 0.60 CFU ± SD/mL and the lowest counts of *E coli* was isolated from Sandwich samples was 2.60 ± 0.20 CFU ± SD/mL. In contrast, *E. coli* was absent in samples of chicken rolls, chicken fry, and milkshakes.

**Table 4:** TCC of ready-to-eat foods collected from Mymensingh district of Bangladesh.

Name of the samples	Number of the samples	TCC (mean log CFU ± SD/mL)
Chicken roll	10	ND
Chicken meat loaf	10	3.57 ± 0.96
Chicken fry	10	ND
Burger	10	3.69 ± 0.08
Pizza	10	4.50 ± 0.60
Sandwich	10	2.60 ± 0.20
Salad vegetables	10	4.09 ± 0.29
Ice-cream	10	2.44 ± 0.25
Yogurt	10	3.14 ± 0.30
Milk shake	10	ND

ND=Not detected, TCC=Total coliform count, CFU=Colonyforming units

### Antibiogram profiles

Antibiotic sensitivity tests of *E. coli* isolates revealed them as sensitive, intermediate, or resistant (Table-5). All 21 isolates were subjected to antibiotic susceptibility test against 12 different antibiotics and it was observed 46.1% were susceptible, 16.6% were intermediate, and 46.1% were resistant to 12 different antibiotics. Among them, 47.6% were MDR among seven antibiotic classes (Table-6). *E. coli* isolated from RTE food samples were resistant to cephalexin, ceftazidime, cefixime, oxytetracycline, and ampicillin, but sensitive to gentamycin, kanamycin, ceftriaxone, colistin, and enrofloxacin. *E. coli* isolates were highly resistant to ceftazidime (95.2%), cefixime (90%), ampicillin (90%) followed by cephalexin (85%), oxytetracycline (61.9%). All isolates of *E. coli* were highly sensitive to gentamycin (85%), kanamycin (80%) followed by ceftriaxone (76.1%) and enrofloxacin (76.1%). This study revealed that RTE foods are significant health hazards due to the presence of MDR *E. coli*.

**Table 5:** Antibiotic susceptibility tests of isolated *Escherichia coli* from ready-to-eat food samples.

Classes of antibiotic	Name of antibiotic disk	Number of isolates sensitive (%)	Number of isolates intermediate (%)	Number of isolates resistant (%)
Penicillin	Ampicillin	2 (9.5%)	-	19 (90%)
Aminoglycoside	Gentamycin	18 (85%)	-	3 (14.2%)
	Streptomycin	8 (38%)	13 (61.9%)	-
	Kanamycin	17 (80%)	-	4 (19%)
Polymyxin-B	Colistin	15 (71.4%)	2 (9.5%)	4 (19%)
Quinolone	Nalidixic Acid	3 (14.2%)	18 (85%)	-
Fluoroquinolone	Enrofloxacin	16 (76.1%)	5 (23.8%)	-
Tetracycline	Oxytetracycline	6 (28.5%)	2 (9.5%)	13 (61.9%)
Cephalosporin	Ceftriaxone	16 (76.1%)	3 (14.2%)	2 (9.5%)
	Ceftazidime	-	0	20 (95.2%)
	Cefixime	-	2 99.5%)	19 (90%)
	Cephalexin	2 (9.5%)	1 (4.7%)	18 (85%)

**Table 6:** MDR profile of *E. coli* isolated from ready-to-eat foods.

*E. coli* isolates (n)	Number of isolates resistant to antibiotic class	MDR profile of *E. coli*	Number of isolates showed resistant (%)	Prevalence of MDR *E. coli* (%)
*E. coli* (21)	19 (1)	Any one of the tested antibiotics (AMP)	0 (0.0)	47.6
	18 (2)	AMP-CN	18 (85.7)	
	10 (3)	AMP-CAZ-O	10 (47.6)	
	5 (4)	AMP-O-CFM-CL	5 (23.8)	
	1 (5)	AMP-O-CN-NA-GEN	1 (4.76)	

AMP=Ampicillin, CN=Cephalexin, O=Oxytetracycline, CAZ=Ceftazidime, CL=Colistin, NA=Nalidixic acid, GEN=Gentamycin, *E. coli=Escherichia coli*, MDR=Multidrug-resistant

### ESBL producing *E. coli*

**Figure-2:**
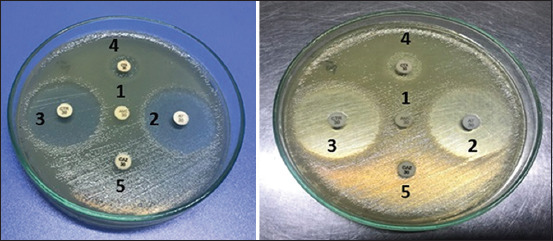
Results of double disk synergistic test, no distortion were recorded with AMC and other antibiotics indicate ESBL negative *Escherichia coli*. 1. AMC=Amoxicillin-Clavulanic acid, 2. CTX=Cefotaxime, 3. AT=Aztreonam, 4. CAZ=Ceftazidime, 5. CTR=Ceftriaxone. ESBL=Extended-spectrum beta-lactamase.

According to 21 isolates, there were no ESBL positive isolates. However, 21 (100%) were ESBL negative (Figure-2 and Table-7).

**Table 7:** Prevalence of ESBL producing *E. coli*.

Number of *E. coli* tested	Number of ESBL positive isolate (%)	Number of ESBL negative isolate (%)
21	0 (0%)	21 (100%)

*E. coli=Escherichia coli,* ESBL=Extended-spectrum beta-lactamase

## Discussion

The availability of fast food (RTE) plays a key role in the lives of city dwellers. If hygienic procedures are not followed during food processing, foods, particularly with fast food, they can carry a number of pathogens and cause a variety of food-borne disorders. Unhygienic and dangerous food handling have a significant impact on public health, resulting in a variety of chronic and non-chronic disorders**.** In Bangladesh, food contamination and food-borne illnesses are very common due to a lack of knowledge, awareness, and adherence to the food laws. *E* *coli* contamination (6.34%) was observed among 101 cooked and prepared food in a University Centre in Argentina [32]. However, the prevalence of *E. coli* was 21% in different RTE food samples collected from different sources in this study. Depending on culture, staining, and biochemical tests, a study in Egypt detected *E. coli* isolates in 12/30 (40%) samples of sausage, 4/30 (13%) of hamburger, 8/30 (27%) of minced beef, and 1/30 (3%) of fried chicken [7]. A study conducted in Tamil Nadu found mixed vegetable salad (80%), panipuri (70%), and veg cutlet (60%) revealed higher positive results for *E. coli*. Whereas the *E. coli* counts in boti masala (80%), chicken tandoori, fish finger, brain masala, and prawn curry (50%) had the highest number of isolates in RTE meat and meat products [33].

A study conducted in Jashore, Bangladesh showed that *E. coli* was isolated phenotypically from 46% of raw salad vegetables and a study in Mymensingh revealed that *E. coli* was isolated from 22% of fresh guava [34, 35] samples. A previous study showed that the highest rate of isolation of *E. coli* was detected in fillets (24%), followed by burgers (12%), then luncheon (8%). In contrast, the examined samples of chicken nuggets were negative for the presence of *E. coli*, with a significant statistical association between the rates of isolation and different poultry products [36]. Our results indicate that there are a high prevalence of *E. coli* in vegetable salad (40%), RTE chicken meat products (20%), and milk products (16.66%) which suggest the production and processing of these foods are not hygienic. The farmers and people involved in every food production and processing stage should be educated about food hygiene. Moreover, another study detected coliform bacteria in 28 (30.4%) of 92 samples, including 7 (28%) of meat meal samples and 3 (20%) of 15 meat-free vegetable meal samples [37]. Another study reported that *E. coli* was detected in 10/275 (3.6%) samples, including 1/65 (1.5%) meat meal samples, 6/79 (7.6%) dessert samples, and 3/90 (3.3%) salad-appetizer samples [38]. Another study on RTE food products containing poultry meat indicated that the incidence of *E. coli* was 17.1%, 14.3%, and 20% of examined samples of chicken shawarma, chicken nuggets, and chicken luncheon, respectively [39]. Furthermore, 27% of RTE cheese samples were found to be contaminated with *E. coli* [40]. The presence of coliforms and *E. coli* in food may indicate fecal contamination and be the result of insufficient cooking, use of raw vegetables, cross-contamination between raw and cooked food and contaminated ingredients. Hence, the presence of *E. coli* found in 21% of the RTE food samples in the present study may likely be the result of fecal contamination.

In this study, all of the ten types of RTE food samples were examined for determination of TCC and most of them were found to be contaminated. TCC in the food items were recorded ranged from 2.60 ± 0.20 log CFU/mL to 4.50 ± 0.60 log CFU/mL. The highest count was estimated from pizza samples (4.50 ± 0.60 log CFU/mL) and lowest count from sandwich samples (2.60 ± 0.20 log CFU/mL). A study in Mymensingh region of Bangladesh [41] estimated the mean TCC of RTE burger samples was 3.28 ± 0.64 log CFU, whereas in our study TCC of burger sample was 3.69 ± 0.08 log CFU; this is essentially similar to results in our study. Another study in Dhaka City, Bangladesh reported *E. coli* in only 2 samples: Faluda and ice-cream, up to 10^2^ CFU/mL [42]. However, in our study, the TCC in ice-cream samples was 2.44 ± 0.25 CFU/mL. In addition, the mean TCC of selected RTE food samples were 2.60 ± 0.20–4.50 ± 0.60, which crossed the acceptable range where the range is 2 log CFU/g [43]. This indicates that a high amount of *E. coli* was present in investigated fast food samples and could cause infections. Similar results were also observed by Somda *et al*. [44], who reported that the mean TCC in grilled, flamed, and fumed chickens found were 2.4 ± 0.82 × 10^7^ CFU/g to 1.27 ± 0.9 × 10^8^ CFU/g. This indicates *E. coli* were present on investigated fast food samples which can induce infection. Similar results were also observed by Nguendo [45], who reported 4.10 × 10^3^ CFU/g in fast food samples in three locations in Yaounde, Cameroon. There was no significant difference (p > 0.05) in the prevalence of *E. coli* across the three different types (milk products, meat products, and salad vegetables) of RTE food samples in this study. The presence of these organisms in RTE food samples is a pointer that these selected RTE food samples were either processed under poor hygienic and sanitary conditions and insufficient processing or could have been from the animal intestines.

The high rate of resistance to more than one antibiotic illustrates increased antibiotic resistance among *E. coli* isolates. The emergence of multidrug resistance in *E. coli* has highlighted the need to raise public awareness, educate physicians and veterinarians, and take appropriate actions to curb indiscriminate antibiotic use. Nearly, 46.1% of the isolated *E. coli* strains were resistant to all the 12 antibiotics, which are quite similar to the findings of Rahman *et al*. [46]. In our study, *E. coli* isolates were highly resistant to ceftazidime (95.2%), followed by cefixime (90%), ampicillin (90%), cephalexin (85%), and oxytetracycline (61.9%). Whereas another study showed that *E. coli* isolated from chicken meat were resistant to oxytetracycline (92%), sulfonamide-trimethoprim (84%), amoxycillin (76%), and erythromycin (60%) [1]. In addition, another study found all *E. coli* strains were resistant to ceftriaxone and cefotaxime and 98% to ceftazidime [24].

Hence, in a therapeutic setting, these drugs should be used with caution and only after antibiotic sensitivity testing. *E. coli* isolated from RTE food samples were highly sensitive to gentamycin (85%), kanamycin (80%) followed by ceftriaxone (76.1%), colistin (71.4%), and enrofloxacin (76.1%) which are supported by the findings of Kumar and Janjir [47]. Another study reported that *E. coli* isolates were resistant to tetracycline (82.4%), which is similar to this study Oje *et al*. [48]. However, another study found 100% of *E. coli were* resistant to ampicillin, amoxycillin, and tetracycline and 50% of *E. coli* were sensitive to gentamicin [40].

Multidrug resistance in *E. coli* has highlighted the need for public awareness, physician education, animal husbandry, and necessary action to be taken. Most of the detected isolates were resistant to all 12 antibiotics. A remarking percentage (47.6%) of the *E. coli* isolates was multi-drug resistance (Table-7). Like-wise a high percentage (73.1%) of the *E. coli* isolates was found to be MDR among 379 isolates [32]. Moreover, a study in China revealed that the *E. coli* isolates showed a high prevalence of resistance to tetracycline (95%), ampicillin (82%), trimethoprim-sulfamethoxazole (78%), nalidixic acid (75%), cephalothin (72%), chloramphenicol (67%), and streptomycin (54%) [49].

## Conclusion

The present study indicates that RTE foods sold in local fast-food shops and bakeries in Mymensingh had unsatisfactory levels of contamination with *E. coli*. In addition, the presence of MDR *E. coli* in these foods may pose serious public health threats. RTE foods vended in Mymensingh represent an unhygienic food preparation practice and reveal the risk factors associated with contamination of post-processing food. To eliminate these risks, one should adopt food and personnel hygiene measures and adequate disinfection and cleaning of equipments and tools used in the kitchen.

## Authors’ Contributions

MMK and MAI: Designed the study. FAE and RNS: Collected and processed the samples for isolation and identification of bacteria. FAE and MZR: Performed PCR and electrophoresis. FAE, MAI, and MMK: Interpreted the results and analyzed the data. FAE, MAI, and MMK: Prepared the manuscript. All authors have read and approved the final manuscript.
